# Authors' Response to Peer Reviews of “Mobile App–Reported Use of Traditional Medicine for Maintenance of Health in India During the COVID-19 Pandemic: Cross-sectional Questionnaire Study”

**DOI:** 10.2196/29626

**Published:** 2021-05-07

**Authors:** N Srikanth, Rakesh Rana, Richa Singhal, Sophia Jameela, Rajeshwari Singh, Shruti Khanduri, Arunabh Tripathi, Sumeet Goel, Leena Chhatre, Ashwin Chandra, B C S Rao, K S Dhiman

**Affiliations:** 1 Central Council for Research in Ayurvedic Sciences Ministry of AYUSH Delhi India; 2 Ministry of AYUSH Government of India Delhi India

**Keywords:** AYUSH Sanjivani app, COVID-19, traditional medicine, Ayurveda, Siddha, Unani, homeopathy

## Round 1:

Thank you for giving us the opportunity to submit a revised draft of our manuscript [1] titled “Utilization of AYUSH Advocacies and Measures in the Indian Population for the Prevention of COVID 19: Insights from a Mobile Application” to JMIR. We appreciate and are grateful for the time and effort that you and the reviewers have dedicated to providing your valuable feedback on our manuscript. We have been able to incorporate the proposed substantial changes to address all the suggestions provided by the reviewers. We are also submitting the previous draft with the amendments made in Track Changes mode for your perusal along with the final “clean” manuscript.

### Reviewer CS:

1. Thank you for pointing this out [2]. We agree with this comment. Therefore, we have deleted the same from the manuscript.
2. You have raised an important point here. Here, the questions in the app were intended to capture whether the use or practice of Ayurveda, Yoga and Naturopathy, Unani, Siddha, and Homeopathy (AYUSH) measures either helped in the prevention of disease or aided in reducing the symptoms of the disease. The purpose of the app was to capture the extent of utilization and acceptance and any benefit that the respondents obtained while using these preventive measures, and we performed a cross-sectional analysis to document and synthesize the same.

In the initial draft, this aspect was not very clear, and in the revised draft, the methodology and the purpose of the app have been elaborated to address this to avoid any misunderstanding.

### Reviewer DL:

1. Agree [3]. It was an inadvertent error. We have modified SARS-CoV to SARS-CoV-2 in the current manuscript.
2. We agree with this, and we have considered this suggestion and have added the screenshot of the welcome page of the mobile app in Multimedia Appendix 1 and the questionnaire in Multimedia Appendix 3.
3. We are thankful to you for pointing this out, and in consideration of your comment, the data sources and data collection methods have been revised to address this issue.
4. We agree with this, and the details of the three modules and layers have been incorporated as Multimedia Appendix 2 for easy understanding.
5. Thank you for this suggestion. We have revised the section and tried to strengthen it with more technical and such other information relevant to the cross-sectional analysis performed on the data documented from the AYUSH Sanjivani app.
6. The tools and methods used for statistical analysis have been incorporated in accordance with your suggestion.
7. We agree that the *Results* and *Discussion* sections were a bit jumbled in the initial version. We have tried our level best to restructure the entire *Discussion* section to avoid redundancies while highlighting and evaluating the results of the cross-sectional analysis.
8. In the app, specific questions were included for the respondents to report whether they have undergone laboratory testing (under medical guidance or by themselves, owing to suspected exposure or due to manifestation of clinical symptoms) and to self-report their disease and symptomatic status.
9. Necessary technical additions and revisions were made to enhance the technical merit of the manuscript.
10. The novelty of this study is that despite being a country with pluralistic health care services and a long history of practice of traditional systems of medicine (Ayurveda, yoga, Unani, Siddha, and homeopathy), the extent of their utilization and acceptability from the perspective of the public has not been explored to date. This is even more relevant because India accounts for more than 17.1% of the total population of the world. However, there are limitations in this study to attain the intended objective, which have been suitably addressed.
11. Proofreading has been done, and identified spelling mistakes and grammatical errors have been rectified.
12. The referencing pattern and style have been changed to comply with the journal standards, and we have incorporated references from recent years.

We look forward to hearing from you in due time regarding our submission and to respond to any further queries and comments you may have.

## Round 2:

We acknowledge the suggestions given by the reviewer and have made the necessary changes in the manuscript.

## Abbreviations

AYUSH: Ayurveda, yoga and naturopathy, Unani, Siddha, and homeopathy
